# Silk Route to the Acceptance and Re-Implementation of Bacteriophage Therapy—Part II

**DOI:** 10.3390/antibiotics7020035

**Published:** 2018-04-23

**Authors:** Expert round table on acceptance and re-implementation of bacteriophage therapy, Wilbert Sybesma, Christine Rohde, Pavol Bardy, Jean-Paul Pirnay, Ian Cooper, Jonathan Caplin, Nina Chanishvili, Aidan Coffey, Daniel De Vos, Amber Hartman Scholz, Shawna McCallin, Hilke Marie Püschner, Roman Pantucek, Rustam Aminov, Jiří Doškař, D. İpek Kurtbӧke

**Affiliations:** 1 Department of Neuro-Urology, Balgrist University Hospital, University of Zürich, CH-8008 Zürich, Switzerland; 2 Nestlé Research Center, Nestec Ltd., Vers-chez-les-Blanc, CH-1000 Lausanne, Switzerland; 3 Leibniz Institute DSMZ-German Collection of Microorganisms and Cell Cultures, D-38124 Braunschweig, Germany; chr@dsmz.de (C.R.); Amber.H.Scholz@dsmz.de (A.H.S.); Hilke.Pueschner@dsmz.de (H.M.P.); 4 Department of Experimental Biology, Faculty of Science, Masaryk University, Brno 611 37, Czech Republic; bardy.pavol@mail.muni.cz (P.B.); pantucek@sci.muni.cz (R.P.); doskar@sci.muni.cz (J.D.); 5 Laboratory for Molecular and Cellular Technology, Queen Astrid Military Hospital, B-1120 Brussels, Belgium; jean-paul.pirnay@telenet.be (J.-P.P.); daniel_de_vos@skynet.be (D.D.V.); 6 School of Pharmacy and Biomolecular Sciences and School of Environment & Technology, University of Brighton, Brighton BN2 4GJ, UK; I.Cooper@brighton.ac.uk (I.C.); J.L.Caplin@brighton.ac.uk (J.C.); 7 Eliava Institute of Bacteriophage, Microbiology and Virology, Tbilisi 0160, Georgia; nina.chanishvili@gmail.com; 8 Department of Biological Sciences, Cork Institute of Technology, Bishopstown, Cork T12 P928, Ireland; aidan.coffey@cit.ie; 9 Department of Fundamental Microbiology, University of Lausanne, CH-1015 Lausanne, Switzerland; shawna.mccallin@unil.ch; 10 School of Medicine & Dentistry, University of Aberdeen, Aberdeen AB25 2ZD, UK; rustam.aminov@gmail.com; 11 GeneCology Research Centre and the Faculty of Science, Health, Education and Engineering, University of the Sunshine Coast, Maroochydore DC, QLD 4558, Australia

**Keywords:** antibiotic resistance, bacteriophages, bacteriophage therapy, Nagoya Protocol, CRISPR CAS

## Abstract

This perspective paper follows up on earlier communications on bacteriophage therapy that we wrote as a multidisciplinary and intercontinental expert-panel when we first met at a bacteriophage conference hosted by the Eliava Institute in Tbilisi, Georgia in 2015. In the context of a society that is confronted with an ever-increasing number of antibiotic-resistant bacteria, we build on the previously made recommendations and specifically address how the Nagoya Protocol might impact the further development of bacteriophage therapy. By reviewing a number of recently conducted case studies with bacteriophages involving patients with bacterial infections that could no longer be successfully treated by regular antibiotic therapy, we again stress the urgency and significance of the development of international guidelines and frameworks that might facilitate the legal and effective application of bacteriophage therapy by physicians and the receiving patients. Additionally, we list and comment on several recently started and ongoing clinical studies, including highly desired double-blind placebo-controlled randomized clinical trials. We conclude with an outlook on how recently developed DNA editing technologies are expected to further control and enhance the efficient application of bacteriophages.

## 1. Introduction

The history of antimicrobial drug discovery includes more than 15 classes of compounds that became a cornerstone in microbial infection control and management and have indisputably saved many lives [1]. Indeed, they have become one of the most successful forms of therapy in clinical medicine. This success, however, is compromised by the emergence and dissemination of antimicrobial resistance, in part due to the widespread (over)use of these compounds in clinical and veterinary medicine and agriculture, thus limiting the efficiency of antibiotics in the control and management of infectious diseases [2]. The extent of the antimicrobial resistance problem in terms of increased morbidity and mortality rates, as well as elevated healthcare costs, has been brought to the public’s attention by several national and international health protection agencies, including the Centers for Disease Control and Prevention (CDC), the World Health Organization (WHO), and the European Medicines Agency (EMA) [3,4,5]. More specifically, WHO resolution 68.7.3 invites international, regional, and national partners to implement the necessary actions in order to contribute to the accomplishment of the five objectives of the global action plan on antimicrobial resistance. If no immediate action is taken, the estimated death toll due to antimicrobial resistance will reach the millions by the year 2050, the cost to the global economy is expected to rise to $100 trillion, and the number of people living in extreme poverty is expected to increase [6]. 

In view of this alarming situation, we published a first opinion paper as a multidisciplinary expert group on the acceptance and re-implementation of bacteriophage therapy in 2016 [7]. In this present perspective paper, we briefly evaluate the status of the previously-made recommendations for bacteriophage therapy over the short term. In addition, we comment on the consequence of the Nagoya Protocol for bacteriophage therapy and then provide an overview on how limitations of the traditional application of bacteriophage therapy could be overcome by the use of Clustered Regularly Interspaced Short Palindromic Repeats/CRISPR associated systems (CRISPR/Cas) gene-edited bacteriophages in the near future.

### 1.1. Factors Impacting the Broad-Scale Application of the Bacteriophages

Four issues have been identified that are limiting, or even preventing, the application of the bacteriophage therapy in the Western World in the 21st century [7]: (1) Quality and quantity of previously conducted study designs, (2) bacteriophage-cocktail production, composition, and application methods in the context of the current legal framework, (3) Lack of awareness among (para-) medical staff and the general public about the potential use of bacteriophage therapy, and (4) Limitations in intellectual property protection for bacteriophage therapeutic applications.

#### 1.1.1. Quality and Quantity of Previously Conducted Study Set-Ups

Bacteriophage therapy has been used for more than 100 years, mainly in Eastern Europe. However, the number of double-blind, placebo-controlled, randomized clinical trials in different fields of medical applications have been limited. Furthermore, they fall far from providing statistically-relevant conclusions about the efficacy of bacteriophage therapy. As a consequence, health authorities and medical professionals in the Western World have been hesitant to proceed with the bacteriophage therapy. Table 1 provides an overview of recently concluded, or currently running clinical studies with bacteriophages with excerpts from https://clinicaltrials.gov/. Notwithstanding the increase in conducted trial numbers, the number of fully-completed and well-documented trials still remains too low to draw substantial conclusions for the diverse range of medical applications where bacteriophage therapy might be implemented. Of the completed trials, several factors have hampered their ability to conclude on the potential efficacy of phage therapy. 

Small patient cohorts and the failure to recruit enough patients have severely limited the conclusions that can be drawn from modern bacteriophage trials. For instance, the recently completed Phagoburn trial, which represented a public investment of 3.85 million euro, enrolled a total of only 27 patients between 11 centers [8,9]. This was far from the pre-calculated 220 patients needed to provide statistically significant results for the study. Reasons cited for the low number of participants were restrictive patient inclusion criteria, a lower incidence of burn wound infections than in previous years, and a shorter recruitment period due to regulatory constraints. Patient enrollment was limited to mono-species infections at the request of the ethical committee, which does not represent the clinical reality of burn-wound infections, and resulted in few eligible patients for the trial. Another trial using bacteriophage for the treatment of pediatric *Escherichia coli* diarrhea also did not reach their estimated patient numbers, because of early trial termination [10]. An in-depth failure analysis of this study revealed that *Streptococcus* sp. may have been a better clinical target than as initially anticipated, *E. coli*.

These two studies, which represent the largest in recent history, highlight several lessons to be learned for future bacteriophage therapy investigations. In addition to individual patient safety, ethical committees should also consider the overarching purpose of clinical trials to produce significant and generalizable results when reviewing clinical trial protocols; the inability to do so is both a detriment to the well-being of society and a waste of trial-eligible patients. Priority should be given to infections with established pathogens, and the test product should reflect the clinical reality to cover indicated pathogens. There is no clinical evidence to suggest a safety concern for targeting multiple pathogens with broad spectrum bacteriophage products, as supported by several recent clinical trials (Table 1), the Polish experience, and the long-standing safe indication for commercial polyvalent bacteriophage cocktails in Russia and Georgia [11,12,13]. Preliminary data from an ongoing trial on patients suffering from urinary tract infections treated with a broad-spectrum bacteriophage cocktail in Georgia will further assuage safety concerns [14]. Furthermore, in this Randomized Clinical Trials (RCT), one of the inclusion criteria requires the in vitro sensitivity of the identified pathogen(s) to the bacteriophage present in the cocktail, thereby acknowledging the very specific bacteriophage-bacterial host interaction. In order to continuously enhance the spectrum of the cocktail during the study, resistant strains are used for adaptation of Pyo bacteriophage cocktail [15].

Given the time and financial investment required for clinical trials, it might be prudent to exploit information from smaller-scale clinical investigations, as well as the ongoing practice of bacteriophage therapy in Eastern countries. Valuable knowledge to understanding bacteriophage therapy has already been generated by The Polish bacteriophage Therapy Unit at the Hirszfeld Institute, a nonprofit entity, that has accumulated data from years of individual patient reports [16]. Good insights can certainly be derived from the several case reports that have provided more in-depth analysis of clinical samples and clinical parameters, even compared to some of the more formal clinical trials. Furthermore, bacteriophage therapy has been approved under emergency treatment schemes in the USA, Australia, France, and Belgium. Table 2 summarizes a collection of recently reported case studies. A thorough and objective assessment on the cost and benefits of bacteriophage production and therapy applications in Russia, Georgia, and Poland, including production protocols, safety, and efficacy, would reveal underlying strategies developed from decades of empirical bacteriophage use. Proper reporting should be a priority for all uses of clinical bacteriophage therapy, whether it be formal trials or case reports [12].

#### 1.1.2. Bacteriophage Production and Application Methods in Context of the Current Legal Framework

Today’s legislation and safety requirements for the production and admission of drugs are, for good reasons, heavily controlled. By having strict quality control procedures, it is assured that drugs are effective, safe, and produced with a consistent quality and composition. The production and application of bacteriophage therapy should not be any different. However, the nature of bacteriophages and their effective medical application are not compatible with current production and admission requirements for chemical drugs. The intrinsic strength of bacteriophages relates to their antagonistic evolution with their bacterial hosts. To assure an effective application of bacteriophage therapy, this requires the ability to continuously adjust and adapt the composition of bacteriophage cocktails. Such a flexible and dynamic production system, coupled with the application of an infection-eliminating medication is incompatible with current legislation and safety requirements set for traditional static and chemically produced drugs. Although the use of bacteriophages is already quite old, in fact, their tailor-made use and applications are in line with the growing demand and insights around personalized nutrition and personalized medicine, where DNA, microbiome composition, and personal lifestyle act as leading indicators. 

Previously, we referred to a couple of proposals to overcome this incompatibility with today’s regulatory frameworks in the Western World, while still assuring safety and efficacy [7]. For instance, the creation of a new EC Directive in Europe concerning bacteriophages and bacteriophage cocktails for human use, or an update of the already existing Medicinal Products Directive 2001/83/EC with a specific amendment for bacteriophages and bacteriophage cocktails, or to register bacteriophages and bacteriophage cocktails under the Council Directive concerning medical devices (93/42/EEC) [32]. A recent breakthrough in this debate has occurred in Belgium, where the national authorities agreed on implementing a pragmatic phage therapy framework that centres on the magistral preparation (compounding pharmacy in the US) of tailor-made phage medicines [33]. There is good reason to believe that this Belgian “magistral phage medicine” framework will be flexible enough to exploit and further explore the specific nature of bacteriophages as co-evolving antibacterials whilst giving precedence to patient safety.

Regarding good manufacturing practices, experts are in agreement that these should be defined in the specific context of bacteriophages as natural entities. The use of whole genome sequencing technologies, together with several additional specific controls [7], can assure the safety of newly identified or adapted bacteriophages and bacteriophage products in general. Rapid sequencing will also allow the safe incorporation of the unique feature of bacteriophage therapy that when in the case of acute infections, new bacteriophages can be isolated within 48 h, or adapted to counteract potential resistance of emerging pathogens. Quality and safety requirements for bacteriophage therapy products have been listed in Table 1 in the previously published paper [7].

Another suggested mechanism that was highlighted by the panel members to ensure the safety of bacteriophage therapy relates to the implementation of a monitoring system. This would function much like that for antibiotic resistance, and should be put into place as soon as bacteriophage therapy has started. The main purpose of the monitoring system is to collect data for prospective analyses, as well as to detect and follow the development of bacterial resistance to bacteriophages. 

Several researchers have made proposals for the safe re-implementation of bacteriophages by establishing validated bacteriophage collections in hospitals for compassionate use applications. In this way, as soon as the bacterial pathogen has been identified, which is often practiced already, it could be tested for sensitivity against such a library of bacteriophages [34]. Another suggestion refers to installing dedicated public structures, National Reference Centres for bacteriophage therapy, that can conduct pilot treatments and facilitate production of hospital-based bacteriophage solutions, and application protocols that will ensure adequate product quality, patient safety and monitoring of treatment efficacy [35]. Such a way of working is, in fact, already operational at the bacteriophage therapy centre located at the Hirszfeld Institute of Immunology and Experimental Therapy in Wroclaw, Poland [16]. 

#### 1.1.3. Lack of Awareness among (Para-) Medical Staff and the Public About Bacteriophage Therapy

For many years, the history and potential of bacteriophage therapy was out of sight for people in the Western World, including medical staff and patients. Following the international increase of antibiotic resistance of pathogens and since the 100th anniversary of bacteriophage therapy in 2017, there has been a clear increase in reporting and communication on bacteriophage therapy in all forms of media in many Western countries, including scientific opinion articles, television programs, and social media initiatives. Also, the increase of medical tourism to Georgia or Poland for bacteriophage treatment illustrates a growing awareness on and demand for bacteriophage therapy. 

Despite this increase of public awareness, panel members feel that there is still not enough interface with the medical community. Little focus is given in the curricula used for (para-) medical trainees on phage therapy, including bacteria-phage antagonistic evolution. Expertise on phage therapy could easily be provided through researchers who by reaching out to their medical colleagues operating at local hospitals. In this way, relevant information can be disseminated about the pros and cons of bacteriophage therapy in the context of the emergence and spreading of antimicrobial resistance. The increased awareness could thus lead to more application of bacteriophages for compassionate use as described above. At the same time, hospitals and patients should be aware that bacteriophage therapy will not always be successful and yet unknown safety risks and complications cannot be excluded.

#### 1.1.4. Limitations in Intellectual Property Protection 

Bacteriophages are ubiquitous natural organisms that are relatively easy to isolate from the environment and have been in the public domain since the 1920s, and therefore the possibilities for intellectual property (IP) protection are limited. Although this might be seen as a limitation by companies intending to seriously invest in bacteriophage therapy, in fact it also offers the opportunity for local, national, and supra-national state-supported medical care to collectively invest in the development of bacteriophage therapy that is expected to be more cost-effective for treatment of many infectious diseases compared to several of the currently used antibiotics. The expected efficacy and affordability of bacteriophage therapy is also illustrated by initiatives around development of bacteriophage therapy in developing countries. In these situations, people are disproportionately impacted by infectious diseases, leading to a critical disease burden on healthcare budgets, and where standard medical care is already difficult to afford for the majority of the population [36]. 

We expect IP protection opportunities to exist for bacteriophage production methods, as well as for applications that can enhance the efficiency and quality of bacteriophage therapy, and/or improve shelf-life stability. Opportunities to protect IP will also come from genetically-engineered bacteriophages, as outlined further in this article. In addition, we see options for private/government partnerships with patent pools under supra national governance that should be managed through organizations such as the WHO, CDC, ECDC or UN [37].

## 2. The Nagoya Protocol and the Implications on Bacteriophage Therapy

In order to increase acceptance and implementation of bacteriophage therapy, it is obvious that rapid and efficient procurement of bacteriophages from the environment with therapeutic potential, and their bacterial hosts, is essential. Bacteriophages, along with all other genetic (biological) resources, are regulated by the Nagoya Protocol (NP). Briefly put, this means that regulations governing the collection of bioresources from natural environments and subsequent benefit-sharing with the country of origin are increasingly important (Box 1). 

In 2017, several authors evaluated the impact of the NP on research and international cooperation, the need for best practices for benefit-sharing, and proposed adjustments to the NP to accommodate microbiological research and development (R&D) [2,3,4]. Since microorganisms are typically ubiquitous and of the same constitution across the world, it was argued that the expectations of the NP as set by lawmakers is scientifically unfeasible. Similar to their bacterial hosts, bacteriophages are also cosmopolitan, and an estimated 10^7^ bacteriophage particles might be present in any one milliliter of natural sample. As a result, thousands of new genetic resources can result from a single step of sampling from the environment, but there are thousands of diverse natural environments around the world that are not limited to one specific country or region of interest. New environmental samples are themselves inherently worthless, as the potential for each bacteriophages and bacterial isolates is unknown and intensive research is necessary to purify and evaluate these characteristics. Additionally, high numbers of specific bacterial hosts are required for phage amplification, to determine the activity spectrum, and to increase their efficacy in clinical applications. Taken together, these scientific realities mean that researchers will be likely to search for bacteriophages/hosts where NP restrictions are either not in place, or where the NP highly efficiently organized. 

Bacteriophages offer great potential for human and veterinary medicine, but NP regulations could be interpreted as in conflict with the WHO objective (and subsequent G7 and G20 summits) calling for all countries to develop alternative antibacterial strategies in human medicine [38]. Also in the case of serious outbreaks of foodborne infections, requiring urgent response, (e.g., Germany in 2011 with the outbreak of *E. coli* O104:H4 EAHEC that caused more than 50 cases of deaths), the NP might impede progress in the global search for potent therapeutics and straightforward exchange of bacteriophages that could save lives.

For microorganisms in general, and for research and applications of bacteriophages in particular, we share the concerns put forward in the Lactic Acid Bacterial Industrial Platform (LABIP) [39] and Microbiological Research Under the Nagoya Protocol: Facts and Fiction [40] and would welcome the following amendments to the NP: 1. Precise definitions of terms like “utilization” and “research and development”, so there is regulatory certainty about what is meant when these words are written in the Nagoya Protocol; 2. Guidelines to consider R&D expenditure around bacteriophages and related investment for basic microbiological research in the terms of agreement on benefit sharing, 3. Simplification of the NP requirements in case of screening activities of a large number of bacteriophages and potential host strains aimed to find a just few candidates with specific characteristics; 4. Research using digital sequence information of bacteriophages and host strains to remain outside the scope of the NP and ABS legislations, as it would be a daunting task to obtain PIC and MAT for all relevant sequences in a database such as GenBank. 5. In case of infection outbreaks, rapid exchange of material should be uncomplicated and governed by a generic international benefit-sharing agreement that is ready for the unexpected.

Box 1 **Explanation about the Nagoya Protocol.**

The NP is a new international regime that came into effect on 12 October 2014, and has been ratified by more than 100 countries. The NP is the implementing treaty for the Convention on Biological Diversity (CBD, www.cbd.int), which itself has been in force since December 1993, and is intended to harmonize access and benefit-sharing mechanisms for the retrieval of biological resources out of provider countries (often in emerging economies). The purpose of the NP is to achieve the objectives of the CBD: 1. Conservation of biodiversity, 2. Sustainable use of the genetic resources and 3. Balanced and equitable sharing of benefits when genetic resources are used (Access and Benefit Sharing (ABS)). Hereto, before starting any research and development work on biological resources, Prior Informed Consent (PIC) by the ‘provider country’ is needed, which done according to Mutually Agreed Terms (MAT) to be laid down in a contract describing access to the materials and how benefits will be shared. In practice, benefit sharing can take a variety of forms, including monetary payments, for example with royalties or research funding, but also via non-monetary forms, such as technology transfer or scientific collaborations. Historically, bioresources were considered a shared heritage of humankind, and the NP allows signatories to regulate access to genetic resources to ensure benefit-sharing with such provider country. Generic guidance flowcharts are given by Overmann and Scholz [40] and Smith et al. [41].

Within the EU, member states are varied in the regulation of access to genetic resources, with northern Member States often allowing unrestricted access and southern States considering regulation. The United States neither ratified the CBD nor the NP, but these international agreements do affect U.S. scientists and aligned standard operating procedures are in development [42]. The primary resource for determining whether there are restrictions imposed on a genetic resource is the ABS Clearing-House (ABSCH) (https://absch.cbd.int/), which provides country profiles and, if appropriate, national regulations that, ideally, explain how to access their sovereign genetic resources, including required documents such as PIC and MAT. Due to the current lack of completed country profiles in the ABSCH, it is often difficult to find the practical information needed to be compliant with the NP. The long-term implications for phage research are unclear, but conceivably threatened.

## 3. The Future of Bacteriophage Therapy by the CRISPR/Cas System

In the era of gene editing, it is relevant to study the potential of genetically-modified bacteriophages as therapeutics. Bacteriophages could be engineered for better efficacy and a broader range of application, and, importantly, would be more attractive for investors due the generation of IP rights (see above). Recent advances in CRISPR/Cas-editing (see Box 2) made bacteriophage DNA editing a hot-topic, since this method can be applied on basically every bacteriophage regardless of its size, host, or properties. Even bacteriophages encoding anti-CRISPR proteins or bacteriophages without a mechanism for repairing CRISPR/Cas-induced breaks can be edited using different CRISPR/Cas types, or by incorporating a repairing protein from another bacteriophage in trans [43]. The strategies based on therapeutic genetically-engineered bacteriophage, which we mentioned previously [7], were constructed almost exclusively for *E. coli*, due to the range of editing strategies already developed for this model bacterium. On the contrary, CRISPR/Cas-editing can be applied also to bacteriophages targeting members of the ESKAPE pathogen group (*Enterococcus faecium*, *Staphylococcus aureus*, *Klebsiella pneumoniae*, *Acinetobacter baumannii*, *Pseudomonas aeruginosa*, and *Enterobacter* species). This has already been proven in *Staphylococcus aureus* and bacteriophage ISP [44], which is member of the same genus *Kayvirus* as a bacteriophage used in the EU-approved therapeutic STAFAL^®^. Thus, previously proposed applications of genetically-engineered bacteriophages are expected to get relevant clinical significance as summarized in Table 3.

Seven bacteriophages from various hosts have recently been successfully edited using three different types of CRISPR/Cas (Table 4): i. type I-E, prevalent in *E. coli*, ii. type II-A, which requires only one protein Cas 9 and sgRNA for editing ability and is best described so far, and iii. type III-A, which does not require a protospacer adjacent motive and cannot be evaded by simple nucleotide substitution [44].

Box 2 **CRISPR/Cas editing.**

CRISPR/Cas (Clustered Regularly Interspaced Short Palindromic Repeats/CRISPR associated systems) was originally identified as a prokaryotic adaptive immune system. It mediates the cleavage of foreign DNA by Cas nuclease if this DNA matches the sequence of spacers in the CRISPR locus. It soon became an important method for eukaryotic genome engineering. However, a limited number of studies have been concerned with its application in bacterial genome engineering due to the poor ability of bacteria to repair CRISPR/Cas-induced breaks [54]. Bacteriophages, on the other hand, have evolved several mechanisms to repair such breaks, as a way to increase the probability of escaping the bacterial immune system. Bacteriophages thus represent an excellent model for optimizing CRISPR/Cas as a prokaryotic genome engineering tool, as well as for various practical applications of modified bacteriophages (for review see Bardy et al. 2016, [55]).

## 4. Limitations and Concerns for CRISPR/Cas Gene Edited Bacteriophages

### 4.1. Efficacy

One of the unknowns concerning CRISPR/Cas-edited bacteriophages relates to whether the mutations will be preserved and reproduced, or if spontaneous mutants that escape the CRISPR/Cas will arise and, due to increased fitness, will outgrow the recombinant. As a consequence, bacteriophages with many point mutations in inserted foreign genes and tags would emerge, rendering the intended modifications useless. A solution could potentially come through the application of CRISPR/Cas III-A, where there has been no evidence of escape mutants [44]. However, in order to understand the long-term effect of CRISPR/Cas-based selections on the bacteriophage population, more research is necessary. In CRISPR/Cas II-A type, it is possible to utilize several spacers targeting different locations in the gene of interest, which will reduce the probability of escape. This technique, however, becomes more laborious with every additional step and might still prove to be non-efficient. 

### 4.2. Legislative Hurdles

CRISPR/Cas-edited bacteriophages with heterologous gene insertions would rank as genetically modified organisms (GMOs), which brings additional hurdles to their approval as therapeutic agents. Their future success will therefore depend on the safety and environmental regulation of GMOs in individual countries. At present, the United States and China appear to have far more amenable policies and prospects for GMO acceptance compared to the EU and Russia. The reproducibility and viability of a genetically modified bacteriophage could also be eliminated by introducing lethal mutations to ensure that the modified bacteriophage would disappear over time, therefore decreasing environmental concerns. Another option is to transform the bacteriophage to a non-replicative delivery vehicle that would mediate killing by an alternative approach (Box 3), which is currently becoming an important focus of research interest in terms of modified bacteriophage therapy. The production of such bacteriophages would require the use of a modified host strain, or presence of a helper bacteriophage to facilitate reproduction. For such applications, the efficacy of such modified constructs in bacteriophage therapy remains to be investigated.

Box 3 **CRISPR/Cas-based antibiotics delivered by bacteriophage.**

Apart from editing, CRISPR/Cas has been utilized as a weapon for the specific killing of virulent or antibiotic-resistant bacterial strains, by reprogramming CRISPR/Cas to target a gene sequence encoding such a property. A bacteriophage can serve as a delivery vehicle, by binding to the relevant bacterial strain and transferring DNA encoding Cas9 nuclease and sgRNA into the cell. This DNA is packaged into the bacteriophage capsids by introduction of specific bacteriophage packaging sites on both ends of the sequence. As a result, only the targeted strain is eliminated, therefore preserving the rest of the microbiome. In addition, the transfer of the undesired phenotypic traits is abolished among strains infected by the delivery bacteriophage. Many groups are studying this strategy (for review see Fagen et al. 2017 [48]), with several start-ups (Locus in US, Nemesis in UK or Eligo in France) already developing such a bacteriophage for commercial use. Still, classic problems of bacteriophage therapy, such as finding suitable bacteriophages for each individual pathogenic strain or the potential immune reaction of the body to large doses of bacteriophage virions, remain obstacles for application.

### 4.3. Safety and Environmental Risks

Despite the absence of adverse reports and also the recent progress and resulting knowledge in the area of natural bacteriophage immunogenicity [16], the mechanism of bacteriophage tolerance by the human immune system is not yet entirely understood. Since bacteriophages need to be applied in a relatively large amount or can replicate at the site of the bacterial infection to high titers, their effect on the human immune system requires thorough investigation. Furthermore, especially in the area of GM bacteriophages, there might be unique health and safety risks that require careful evaluation, such as the possible side effects around the application of long-circulating bacteriophages (Table 3). Also, bacteriophages with modified structural proteins, displaying purification tags, or with additional receptor-binding domains may represent novel antigens for the immune system. In the case of using bacteriophages as delivery vehicles, even in the non-lytic killing strategy, cells could be lysed by the patient’s immune system and the cell content containing modified proteins or endotoxins thus released into the bloodstream. The (in) stability of modified bacteriophages and the consequences of spontaneous mutations are other safety targets that require investigation. 

Given the continuous need for propagating host strains, the probability of survival of bacteriophages, including modified bacteriophages, outside the laboratory or hospital is minimal. However, the risk of recombination between modified and wild-type bacteriophages or horizontal gene transfer among modified bacteriophages and bacteria must be evaluated, particularly in case bacteriophages would carry genes foreign antibacterial products. Theoretically, uptake and incorporation of such sequences by a bacterium may result in a fitness advantage over the strain for which the bacteriophage was originally designed. 

## 5. Discussion and Conclusions

The urgent need for effective alternatives to antimicrobials is self-evident in order to reduce the morbidity and mortality associated with antimicrobial resistance, as well as reducing the healthcare burden on economies. Many proposals have been made on which path bacteriophage therapy should follow in order to be approved. A recurrent conclusion is that this therapy will never become a viable option if we continue assessing this treatment under the same regulatory frameworks as for chemical drugs. In fact, bacteriophage therapy is already safely being applied today, as demonstrated not only from ongoing activities in Eastern Europe, but also from the observation that already for more than a decade several of the successful bacteriophage therapy cases reported in the Western World (Table 2) are led by military hospitals who have the flexibility to invest in therapies which are not yet legally approved. 

If bacteriophage therapy is to develop as a means to stop the emergence of antibiotic resistance, courageous decision making is needed that allows the controlled use of bacteriophage. This needs to take into consideration the many years of practice in Russia, Georgia and Poland, and which is in line with many good proposals from scientists and physicians who understand the specific nature of bacteriophages. 

At the same time, we should not expect that bacteriophage therapy will always be 100% effective and might occasionally induce side effects, as we will understand better upon the implementation of bacteriophage therapy monitoring systems. But how does the occurrence of potential side effect compare to the opportunity costs of not applying bacteriophage therapy in case of antibiotic resistance? Furthermore, also for antibiotics that in many cases are lifesaving, we accept undesired side effects, for instance when due to the unspecific mode of action of antibiotics beneficial microbes are also affected, which often causes complications such as antibiotic-induced dysbiosis and secondary infections. 

A great step forward can be made when more countries will follow the example of the Belgian Ministry of Public Health regarding the set- up of a phage therapy framework that centers on the magistral preparation (compounding pharmacy in the US) of tailor-made phage medicines. Importantly, this Belgian solution assures safety and avoids the application of certain medicinal product requirements that restrain flexible phage therapy approaches, such as compliance to Good Manufacturing Practice [33]. 

In this paper, we have underlined the call for more RCTs with bacteriophage therapy. In this context, it is also an important responsibility of the researchers involved to properly report the outcome of the studies, as has not always been the case (Table 1). Incomplete reporting on clinical studies does not help to gain confidence on pros and cons of new therapies. Besides the mentioned need for more studies, at the same time we reason that depending on the type of infection, a traditional RCT might not always be the most efficient route to determining bacteriophage efficacy, and it is not only bacteriophage therapy that finds itself in this predicament. An increase in personalized-medicine approaches, particularly for cancer treatments, have necessitated different designs for clinical trials [56]. N-of-1 studies, where a patient is the entire study, has become increasingly frequent to evaluate treatments for rare diseases or pain reducers. Basket designs permit the selection of patients with molecular markers that make them likely to respond to a certain treatment; this is not unlike pre-testing bacterial isolates for bacteriophage sensitivity prior to trial enrollment. The increasing frequency of case-reports for bacteriophage therapy suggests that bacteriophage therapy may be heading in the direction of a personalized treatment, at least for some diseases (Table 2). 

With regard to the Nagoya Protocol, we call for pragmatic approaches to obtain Prior Informed Consent (PIC) and Mutually Agreed Terms (MAT) that are proportional to the value of the immediate foreseen benefits. Otherwise, due to the delays that might be encountered in the process, there may not be any significant benefit to share. Since there are, at present, very few culture collections with bacteriophage holdings, new bacteriophages have to be isolated in high numbers to constantly fulfill the WHO resolution 68.7.3 (WHO Assembly October 2016). The proposed amendments and clarifications to NP as mentioned in the section on NP could facilitate that the protocol does not unintendedly retard these innovative developments that are important for humankind in the 21st century. This is especially valid for a rapid and effective application of bacteriophages in the events of emergency since users should ensure legally-complied uses of the genetic resources, including those for collaborative projects. 

While it becomes increasingly acknowledged that bacteriophage therapy, as applied already for many years in some countries, has great potential to combat the consequences of increasing antibiotic resistance, we acknowledge that the traditional bacteriophage therapy also has some limitations. In this article, we have described that the use of CRISPR/Cas to specifically edit the genome of natural bacteriophages offers an opportunity for controlled and effective bacteriophage therapy and may facilitate reliable future applications of the bacteriophage therapy. However, today several technical issues and safety concerns of GM bacteriophages still need to be addressed. The first step would be to prove that modified bacteriophages could be prepared in large-scale, without any significant background of undesirable mutants. Next, research should focus on immunogenicity assessment of modified virions and potential horizontal transfer of genes encoding antibacterial products. We expect that in a time frame of five-to-ten years, it will become clear if this approach for bacteriophage therapy is worthwhile. Furthermore, it is foreseen that CRISPR/Cas-edited bacteriophages will revolutionize bacteriophage therapy, if not by direct application of modified bacteriophages, by the enormous possibilities that it will bring to fundamental research on bacteriophage biology.

Although the entire world seems to agree on the clear urgency to implement novel solutions, it is worrying to see that therapy with natural bacteriophages has yet to be seriously considered in the Western World. Notwithstanding some open questions, there are many realistic options and scenarios that would enable a gradual, pragmatic, and responsible re-implementation of bacteriophage therapy in the short term to help avoid needless deaths due to antibiotic-resistant infections. Any further delay could be seen as a supported prolongation of the difficult conditions many patients without alternative treatment options find themselves in today.

## Figures and Tables

**Table 1:** Overview of bacteriophage therapy clinical studies.

Name of Study and Organizations Running the Study	Target Organism(s)	Description and Objectives	Outcome Measures	Additional Comments	Clinical Trials.gov Identifier/Reference
Bacteriophages for treating urinary tract infections in patients undergoing transurethral resection of the prostate: a randomized, placebo-controlled, double-blind clinical trial. Tzulukidze National Center of Urology, Tbilisi, Georgia; Eliava Institute of Bacteriophages, Microbiology, and Virology in Tbilisi, Georgia; Balgrist University Hospital, Zürich, Switzerland The study is run in The Republic of Georgia.	*Enterococcus* spp., *Streptococcus* spp., *Escherichia coli*, *Proteus* spp., *P. aeruginosa*	Randomized placebo-controlled double-blind clinical trial: Patients planned for transurethral resection of the prostate are screened for UTIs and enrolled if eligible microorganisms in urine culture are ≥10^4^ cfu/mL. Patients are randomized in a double-blind fashion to the three study treatment arms of 27 people, each in a 1:1:1 ratio to receive either: (a) bacteriophage (b) placebo solution, or (c) antibiotic treatment according to the antibiotic sensitivity pattern.	Primary: Success of intravesical treatment, defined as normalization of urine culture (no evidence of bacteria, i.e., <10^4^ colony forming units/mL) after 7 days of treatment. Secondary: Adverse events, in categorization according to the National Cancer Institute Common Terminology Criteria for Adverse Events (CTCAE) version four in grade one to five. Tertiary: Changes in bladder and pain diary assessment of number of voids, number of leakages, post void residual.	The study uses the commercially available Pyo-bacteriophage cocktail as produced by The Eliava institute in Tbilisi. 81 patients are involved. The Pyo-bacteriophage cocktail is subjected to continuous adaptation during the study. The study started in 2016, and is expected to end in 2018.	NCT03140085 [14]
PHAGOPIED - Standard treatment associated with bacteriophage therapy vs. placebo for diabetic foot ulcers infected by *Staphylococcus aureus*. The study is run by the Centre Hospitalier Universitaire de Nīmes, France, with collaboration with Pherecydes Pharma, Romainville, France.	*Staphylococcus aureus*, MSSA and MRSA	This project utilizes anti-*Staphylococcus* bacteriophages, delivered topically, vs. a placebo control. The study uses random allocations in a parallel assignment intervention design. The main objective of this study is to compare the efficacy of standard treatment associated with a topical anti-staphylococcal bacteriophage cocktail versus standard treatment plus placebo for diabetic foot ulcers mono-infected by methicillin-resistant or susceptible *S. aureus* (MRSA or MSSA) as measured by the relative reduction in wound surface area (%) at 12 weeks.	Primary: The relative reduction in wound surface area over 12 weeks. Secondary: Safety effects, local side effects (rash onset or worsening of local inflammatory signs) and general symptoms (vital signs, fever, rash, arthralgia, gastrointestinal symptoms).	First posted online in 2016. This study is not yet recruiting, but 60 patients are expected to join.	NCT02664740
MUCOPHAGES - Bacteriophage effects on *Pseudomonas aeruginosa.* The study is run by the University Hospital Montpellier, France.	*Pseudomonas aeruginosa*	The study is designed to evaluate the efficacy of bacteriophages on *P. aeruginosa* isolates recovered from sputum.	The study utilizes a suspension of ten bacteriophages. These are tested against isolates recovered from cystic fibrosis patients, to determine their ability to infect these strains.	Completed in 2012. No results posted online.	NCT01818206 [17]
Experimental bacteriophage therapy of bacterial infections. The study is led by the Polish Academy of Sciences.	*Staphylococcus*, *Enterococcus*, *Pseudomonas*, *Escherichia*, *Klebsiella*, *Proteus*, *Citrobacter*, *Acinetobacter*, *Serratia*, *Morganella*, *Shigella*, *Salmonella*, *Enterobacter*, *Stenotrophomonas*, *Burkholderia*	The study uses suspensions of lytic bacteriophages active against clinical isolates of the test species. The program determines to use bacteriophage treatment in a therapeutic role where no other viable treatment is available. For each patient only, specific formulations of single bacteriophage or a bacteriophage mixture that are active against the pathogenic bacterial strain or strains isolated from the patient are used for the treatment (oral, rectal and/or topical application).	The principle focus of the work is to use bacteriophage suspensions to treat the following conditions: bone, upper respiratory, genital and urinary tract infections, as well as post-operative non-healing wounds where antibiotic treatment has not produced positive results.	Start: 2005. Current status unknown. Last update posted in 2013. Number of persons involved has not been stated.	NCT00945087 [11]
PhagoBurn. Phase I/II Clinical Trial. This Project is a European Research & Development (R&D) Project Funded by the European Commission Under the 7th Framework Program for Research and Development Involving seven Clinical Sites in France, Belgium & Switzerland.	*Escherichia coli* & *Pseudomonas aeruginosa*	Evaluation of bacteriophage therapy for the treatment of *Escherichia coli* and *Pseudomonas aeruginosa* wound infections in burned patients A randomized, parallel assignment study assessing tolerance and efficacy of local bacteriophage treatment of wound infections due to *E. coli* or *Ps. aeruginosa* in burned patients.	This study tests the efficacy of *E. coli* and *Ps. aeruginosa* bacteriophage cocktails against silver sulfadiazine to treat wound infections by those bacterial species. Primary: Time necessary for a persistent bacteria reduction of two modes or persistent bacteria eradication relative to D0 adjusted on antibiotic treatment (active on targeted strain) introduced between D1 to D7. Secondary: Assessment of tolerance of treatment over 21 days. Adverse events frequencies will be assessed in each treatment arm. bacteriophage therapy safety profile will be compared to safety profile of standard of care. Incidence and delay of infection reduction with different bacterial species from the targets over a period of seven days. Number of sites cured: The number of infected burns or infected wounds getting a clinical improvement will be described and compared between treatment group over a period of 7 day.	Launched in 2013 and achieved in 2017, PhagoBurn was the world first prospective multicentric, randomized, single-blind and controlled clinical trial of bacteriophage therapy ever performed according to both Good Manufacturing (GMP) and Good Clinical Practices (GCP). Only 27 patients between 11 centers were included, which is far from the pre-calculated 220 patients needed to provide statistically significant results for the study. See main text for more information and lessons learned.	NCT02116010 [8,9]
Antibacterial treatment against diarrhea in oral rehydration solution. The study was run by Nestlé, Switzerland in collaboration with Dhaka Hospital of the International Centre for Diarrheal Disease Research, Bangladesh.	*Escherichia. coli* (T4 bacteriophage)	This randomized double-blind, placebo-controlled trial aims to demonstrate the potentials of a new form of therapy for childhood diarrhea, by measuring the effect of oral administered E. coli bacteriophage in children aged 4–60 months of age with proven ETEC and EPEC diarrha.	Primary outcome measures: Assessment of safety, tolerability and efficacy (reduce severity of diarrhea assessed by reduced stool volume and stool frequency) of oral administration of T4 bacteriophages in young children with diarrhea due to ETEC and/or EPEC infections. Time frame: five days. Secondary outcome measures: Clinical assessment, blood tests, morbidity, duration of hospitalization. Time frame: five days.	First posted online 2009; study ended in 2013. Oral coliphages showed a safe gut transit in children, but failed to achieve intestinal amplification and to improve diarrhea outcome, possibly due to insufficient bacteriophage coverage and too low *E. coli* pathogen titers requiring higher oral bacteriophage doses.	NCT00937274 [10]
Existence in the human digestive flora of bacteriophages able to prevent the acquisition of multiresistant Enterobacteria (PHAGO-BMR). The study is led by Assistance Publique-Hôpitaux de Paris, France.	MDR- Enterobacteria	The study plans to recruit 460 people hospitalized in intensive care unit (resuscitation). The choice of this unit is linked to the fact that the monitoring of resistant bacteria is carried out regularly during the hospitalization. On stool samples collected at separate times of the stay (admission and then during the stay), the scientists look for 2 types of bacteria and viruses capable of destroying them.	Primary: Presence or absence of bacteriophages capable of lysing circulating Ec-ESBL/EPC or Kp-ESBE/EPC in resuscitation units in non-carriers having acquired carriers *E. coli* or *K. pneumoniae* producing ESBL or carbapenemases. Secondary: Presence or absence of bacteriophages in patients identified as carriers of Ec-ESBL/EPC or Kp-ESBL/EPC at entry to resuscitation (control population). Isolated bacteriophages will be characterized.	First posted online in 2017. The study is not yet recruiting.	NCT03231267
METAKIDS Phages dynamics and influences during human gut microbiome establishment.	Enteric microbial species	This project relies on the ability of Meta3C, a technique developed to identify the bacterial host genomes of the different bacteriophages the investigators will detect thanks to the physical collision these molecules experience. Given the role that human gut bacteriophages may play in shaping the development of host microbiomes, their potential for application is of great interest.	Primary outcome measures: Genomic reconstruction and characterization of the different genomes (phages, bacteria, yeast) present in the human gut during the three first years of life. These outcomes will provide a large catalog of DNA sequences. Characterization of the variation of the different species present in human gut during the three first years of life and characterization of bacteriophages-bacteria interactions. This outcome will provide access to the dynamics of the different species present during this period and possibility to correlate them with environmental variation (dietary, age). Secondary: Characterization of bacteriophages and bacteria variations in response to environmental perturbations during infant gut development. Time frame: two to three weeks.	First posted online in 2017; the study is currently recruiting. Estimated enrollment is 20 persons.	NCT03296631
Evaluate bacteriophage as a useful immunogen in patients with primary immune deficiency diseases (PIDD) The study is run by the University of South Florida, USA.	*Escherihia. coli*	This protocol is designed to ascertain whether the bacteriophage 0X174 neoantigen is safe and effective as an antigen used in the evaluation of primary and secondary immune responses. Bacteriophage 0X174 is given intravenously two billion PFU/Kg of body weight; small blood specimens of 3–5 mL (about 1 teaspoon) are collected after 15 min, 7 days, 14 days, and 28 days.	Primary Outcome Measures: Evidence of capacity of switch from IgM to IgG during 12 weeks of trial. Blood samples are obtained after each immunization of bacteriophages.	Current status unknown. First posted online in 2012. Last update 2012. All patients receive two doses of bacteriophages. Selected patients may receive a tertiary vaccine.	NCT01617122
Evaluation and detection of facial *Propionibacterium acnes* bacteria and bacteriophage The study is led by Maccabi Healthcare Services, Israel.	*Propionibacterium acnes*	This multi-center, outpatient study will extract and evaluate the presence of facial *P. acnes* bacteria and bacteriophage strains using pore strips on up to 400 human subjects. An additional *P. acnes* visual detection method (VISIOPOR® PP34N) will be used in this study as per PI decision to explore whether there is a correlation between *P. acnes* bacterial presence and fluorescent signal.	Primary: Detection and analysis of facial *P. acnes* presence. Time frame: Day 0 and week 8. Secondary: Assessing correlation between bacteriophage and *P. acnes* using a. Demographic Questionnaire b. Visual Supportive Methodology (VISIOPOR® PP34N) as per PI decision. Time frame: As above.	First posted online in 2017. Not currently recruiting, but 400 people are estimated to participate.	NCT03009903
Bacteriophages PreforPro cocktails as novel Prebiotics The study is led by Colorado State University, USA. PreforPro is commercialized by Deerland Enzymes, Kennesaw, GA, USA	Enteric bacteria	The bacteriophage Study is a randomized, double-blind, placebo-controlled crossover trial that investigates the utility of four supplemental bacteriophage strains (LH01-Myoviridae, LL5-Siphoviridae, T4D-Myoviridae, and LL12-Myoviridae) to modulate the gut microbiota, and therefore ameliorate common inflammation-related GI distress symptoms (e.g., gas, bloating, diarrhea, constipation, etc.) experienced by healthy individuals. The main goal of this study is to see if consumption of PreforPro, a commercially available prebiotic dietary supplement consisting of a mixture of bacteriophages, improves gut bacteria profiles in individuals relative to a placebo control.	Primary: Microbiota modulation. Time frame: Baseline visit prior to starting treatments, four weeks after starting treatment one, end of two-week washout period, 4-weeks after starting treatment two. Use of 16s rRNA sequencing of stool samples to determine whether the administered interventions resulted in changes to microbial composition. Secondary: Local inflammation Time frame: as above. Inflammation in the bowels will be assessed by use of ELISA test for fecal calprotectin. Systemic Inflammation. Time Frame: As above. Systemic inflammation will be assessed by an ELISA test for CRP and circulating cytokines and immune factors.	The study completed in 2017, but results have not yet been posted online. 43 persons enrolled in the study.	NCT03269617
The Use of Bacteriophage Phi X174 to Assess the Immune Competence of HIV-Infected Patients in vivo. The study is run by the National Institute of Allergy and Infectious Diseases, USA.	*Escherichia. coli*	The objective of this study is to evaluate the safety and utility of bacteriophage phi X174 immunization as a tool to assess the immune competence of HIV-infected patients at different stages of disease in vivo, and to assess the impact of viral load levels and therapy-induced changes in viral load levels on the response to immunization with the neo-antigen bacteriophage phi X174.	Primary. Immune parameters (not further published online.	Study started in 1996, and ended in 2000. 52 patients were involved in the study.	NCT00001540 [18]
Randomized and double-blinded placebo-controlled study of topical application of AB-SA01 cocktail to intact skin of healthy adults. The study is run by AmpliPhi bacteriophage Ltd., the US Army, and the Walter Reed Army Institute of Research Clinical Trials Center, USA.	*Staphylococcus aureus*	The study aims to examine the safety of ascending doses of AB-SA01 when topically applied to intact skin of healthy adults. AB-SA01 consists of three bacteriophages (viruses) that target *Staphylococcus aureus* bacteria. The safety of AB-SA01 will be assessed when topically administered once daily to the volar aspect of the forearm at different doses for three consecutive days.	Primary: Occurrence, intensity, and relationship of adverse events (AEs) from first dose through the end of study visit (14 ± 2 days). Change from baseline in clinical laboratory tests. Time frame: Day 0 (pre-dose), Day 3, and Day 14 ± 2 days. Clinical laboratory tests (hematology, chemistry, and urinalysis). Skin Reaction change from Baseline. Time Frame.	First published online in 2016, and the last update was in 2016 as well. 12 persons recruited.	NCT02757755
A prospective, randomized, double-blind controlled study of WPP-201 for the safety and efficacy of treatment of venous leg ulcers. The study was run by Southwest Regional Wound Care Center, USA.	*Pseudomonas aeruginosa*	The study was designed to assess the safety of *Ps. aeruginosa*-specific bacteriophages for the treatment of leg ulcers in human patients. WPP-201 is a pH neutral, polyvalent bacteriophage preparation, which contains 8 bacteriophages lytic for *Ps. aeruginosa*, *S. aureus*, and *E. coli*. The cocktail contains a concentration of approximately 1 × 10^9^ PFU/mL of each of the component monophages.	Primary: Evaluate the safety of the use of WPP-201.	Study started in 2008 and was completed in 2011. 64 patients were involved. This study found no safety concerns with the bacteriophage treatment. Efficacy of the preparation will need to be evaluated in a phase II efficacy study.	NCT00663091 [19]

**Table 2:** Human bacteriophage therapy related case studies published in peer-reviewed English-language scientific literature over the last ten years.

Case Study Title	Description	Outcomes	Comments	Reference
Refractory *Pseudomonas* Bacteremia in a Two-Year-Old sterilized by bacteriophage therapy	The authors report a complex case that involved a pediatric patient who experienced recalcitrant multidrug-resistant *Pseudomonas aeruginosa* infection complicated by bacteremia/sepsis; antibacterial options were limited because of resistance, allergies, and suboptimal source control.	A cocktail of 2 bacteriophages targeting the infectious organism introduced on 2 separate occasions sterilized the bacteremia.	USA	[20]
Development and use of personalized bacteriophage-based therapeutic cocktails to treat a patient with a disseminated resistant *Acinetobacter baumannii* infection	The authors report on a method used to produce a personalized bacteriophage-based therapeutic treatment for a 68-year-old diabetic patient with necrotizing pancreatitis complicated by an MDR *A. baumannii* infection. Despite multiple antibiotic courses and efforts at percutaneous drainage of a pancreatic pseudocyst, the patient deteriorated over a 4-month period. In the absence of effective antibiotics, two laboratories identified nine different bacteriophages with lytic activity for an *A. baumannii* isolate from the patient.	Administration of bacteriophages intravenously and percutaneously into the abscess cavities was associated with reversal of the patient’s downward clinical trajectory, clearance of the *A. baumannii* infection, and a return to health.	USA	[21]
Phage therapy in a 16-year-old boy with Netherton syndrome	The authors report on a 16-year-old male with all the typical manifestations of Netherton Syndrome, including atopic diathesis and ongoing serious staphylococcal infections and allergy to multiple antibiotics whose family sought help at the Eliava bacteriophage Therapy Center when all other treatment options were failing.	Treatment with several antistaphylococcal bacteriophage preparations led to significant improvement within seven days and very substantial changes in his symptoms and quality of life after treatment for six months, including return visits to the Eliava bacteriophage Therapy Center after three and six months of ongoing use of bacteriophage at home	Georgia. (Patient came from France)	[22]
Use of bacteriophages in the treatment of colistin-only-sensitive *Pseudomonas aeruginosa* septicaemia in a patient with acute kidney injury—a case report	A 61-year-old man with gangrene of the peripheral extremities, resulting in the amputation of the lower limbs and the development of large necrotic pressure sores, developed septicaemia with colistin-only-sensitive *P. aeruginosa*. Intravenous colistin therapy was started. Ten days later, the patient developed acute kidney injury and antibiotic therapy was discontinued to prevent further kidney damage. *P. aeruginosa* septicaemia re-emerged and two bacteriophages, which showed in vitro activity against the patient’s *P. aeruginosa* isolates, were administered as a 6-h intravenous infusion for ten days.	Immediately upon bacteriophage application, blood cultures turned negative, CRP levels dropped and the fever disappeared. Kidney function recovered after a few days. Hemofiltration was avoided and no unexpected adverse events, clinical abnormalities or changes in laboratory test results that could be related to the application of bacteriophages were observed.	Belgium The patient died four months after bacteriophage therapy of sudden in-hospital refractory cardiac arrest due to blood culture-confirmed *Klebsiella pneumoniae* sepsis.	[23]
Bacteriophage treatment of intransigent diabetic toe ulcers: a case series	The authors present a compassionate-use case series of nine patients with diabetes and poorly perfused toe ulcers containing culture-proven *Staphylococcus aureus* infected bone and soft tissue, who had responded poorly to recommended antibiotic therapy.	All infections responded to the bacteriophage applications and the ulcers healed in an average of seven weeks with infected bone debridement. One ulcer, where vascularity was extremely poor and bone was not removed to preserve hallux function, required 18 weeks of treatment.	USA	[24]
Use of bacteriophages in the treatment of *Pseudomonas aeruginosa* infections	The author reports on bacteriophage treatment of *Pseudomonas aeruginosa* otitis in a pet dog and in a human burn wound patient.	Symptomatic improvement and bacteriophage multiplication were seen in the pet dog and in the human patient.	UK	[25]
Clinical aspects of bacteriophage therapy	The authors present a detailed retrospective analysis of the results of bacteriophage therapy of 153 patients with a wide range of infections resistant to antibiotic therapy admitted for treatment at the bacteriophage therapy unit of the Ludwik Hirszfeld Institute of Immunology and Experimental Therapy, Wrocław, Poland, between January 2008 and December 2010.	Data suggest that bacteriophage therapy provided good clinical results in a significant cohort of patients with otherwise untreatable chronic bacterial infections and is essentially well tolerated.	Poland	[11]
Bacteriophage therapy for refractory *Pseudomonas aeruginosa* urinary tract infection	The authors describe adjunctive bacteriophage therapy for refractory *Pseudomonas aeruginosa* urinary tract infection in the context of bilateral ureteric stents and bladder ulceration, after repeated failure of antibiotics alone.	Combined therapy was well-tolerated, apparently resulting in symptomatic relief and microbiological cure where repeated courses of antibiotics combined with stent removal had failed. Bacteriophage did not persist nor was any antibiotic- or bacteriophage resistant *P. aeruginosa* identified.	Australia Additive effort of combined bacteriophage-antibiotic treatment	[26]
Eradication of *Enterococcus faecalis* by bacteriophage therapy in chronic bacterial prostatitis	The authors report on the treatment of three patients suffering from chronic bacterial prostatitis who were qualified for an experimental bacteriophage therapy protocol managed at the bacteriophage Therapy Unit in Wrocław. The patients had previously been treated unsuccessfully with long-term targeted antibiotics, autovaccines, and laser biostimulation.	Rectal application of bacteriophage lysates targeted against *Enterococcus faecalis* cultured from the prostatic fluid gave encouraging results regarding bacterial eradication, abatement of clinical symptoms of prostatitis, and lack of early disease recurrence.	Poland	[27]
Corneal Infection Therapy with Topical Bacteriophage Administration	A 65-year-old woman suffering from MDR *S. aureus* infection of the cornea as a post-operative complication of a craniotomy. This patient suffered the chronic, persistent infection for years before seeking therapy in Tbilisi, Georgia. A single bacteriophage was administered both topically in the eye and nasal application and intravenous application for four weeks.	The patient’s regular physicians published that the patient’s ocular and nasal cultures after returning from Georgia were negative at three and six months post-treatment.	Georgia. (Patient came from France)	[28]
The use of a novel biodegradable preparation capable of the sustained release of bacteriophages and ciprofloxacin, in the complex treatment of multi-drug resistant Staphylococcus Aureus-infected local radiation injuries caused by exposure to Sr90.	Authors report the topical use of PhagoBioDerm (phage + ciprofloxacin wound polymer) to treat two wounds infected with *S. aureus* after the failure of conventional treatment. The responsible pathogen was resistant to ciprofloxacin, but sensitive to the bacteriophage.	PhagobioDerm resulted in reduced purulent drainage and symptom amelioration. *S. aureus* was eliminated from the wound	Georgia	[29]
Successful eradication of methicillin-resistant *Staphylococcus aureus* (MRSA) intestinal carrier status in a healthcare worker	Healthcare worker suffered urinary tract infections caused by MRSA that was carried in the GI tract. Authors report that bacteriophage was applied orally	Eradiation of carrier status	Poland	[30]
Phage therapy compassionate use in France in 2017.	Abstract presented by Pherecydes at bacteriophages-sur-Yvette in November 2017 documenting the use of bacteriophage to treat two patients with severe bone and joint infections caused by MDR organisms with topical bacteriophage at a hospital in Lyon.	Symptom amelioration and no reported side effects.	France	[31]
Open-label treatment of RCT with bacteriophages for treating urinary tract infections in patients undergoing transurethral resection of the prostate: a randomized, as mentioned in Table 1.	Prior to the start of the double-blind placebo-controlled trial, nine patients were treated with bacteriophage Pyo cocktail.	In six of nine patients, the titer of the pathogenic bacteria was decreased, varying between 1 log and 7 log (sterile).	Georgia	NCT03140085 [14].

**Table 3:** Targets for genetic engineering for bacteriophage therapy applications.

Gene	Modification	Purpose	Advantage to Natural Bacteriophage Therapy	Related Reference
Antimicrobial protein *	Insertion/gene replacement	Phage killing other strains	Product for mixed infections	[7]
Biofilm degrading enzyme *	Insertion/gene replacement	Degrading biofilm	More active against biofilm-producing strains	
Virulence factor	Gene deletion	No virulence transfer	Novel therapeutic bacteriophage	
Baseplate proteins/tail fibers	Gene replacement	Altered host-range	Novel therapeutic bacteriophage for mixed infections	
Receptor-binding protein/structural proteins	Single gene mutations	Broader host-range	More effective, and faster to obtain than by natural selection	[45]
Major capsid protein	Purification tags insertion	More efficient purification	Purer product	[46]
Major capsid protein	Anti-immune tags insertion/single gene mutations	Longer circulation in bloodstream	More effective, and faster to obtain than by natural selection	[47]
Various, e.g., lytic module	Gene knockout	Non-replicative bacteriophage	Replication control of a bacteriophage	[48]
Endotoxin antibody *	Insertion/gene replacement	Endotoxin removal	Safer product	[49]

* Foreign genes.

**Table 4:** Summary of Clustered Regularly Interspaced Short Palindromic Repeats/CRISPR associated systems (CRISPR/Cas) applications in the editing of bacteriophages.

Bacteriophage	Host	CRISPR/Cas Type Employed	Mutation	Purpose	Gene	Reference
T7	*E. coli*	I-E	Gene deletion	PoC of CRISPR/Cas-editing in bacteriophage	gene 1.7	[43]
2972	*Streptococcus thermophilus*	II-A/Cas9	Gene replacement with methyltransferase/Gene deletion	PoC of native II-A editing, bacteriophage resistant to RM system	orf 33, 39	[50]
ICP1	*Vibrio cholera*	I-E	Gene deletion/Gene replacement with GFP	PoC in *Vibrio* model	*cas* 1, *cas* 2–3	[51]
P2	*Lactococcus lactis*	II-A/Cas9	Deletion/insertion/substitution	PoC of heterologous II-A editing	orf 24, 42, 47, 49	[52]
T4	*E. coli*	II-A/Cas9	Substitutions, deletion	PoC of editing by two gRNA, functional study of gene knock-out	*mcp*, *rnl*B	[53]
Andhra	*Staphylococcus epidermidis*	III-A/Cas10	Substitutions	PoC of native III-A editing	orf 9, 10	[44]
ISP	*Staphylococcus aureus*	III-A/Cas10	Substitutions	PoC of heterologous III-A editing	orf 61	[44]

PoC—proof-of-concept, RM—restriction modification.
